# Canal Transportation and Centering Ability of Twisted File and Reciproc: A Cone-Beam Computed Tomography Assessment

**Published:** 2014-07-05

**Authors:** Kiumars Nazari Moghadam, Shahriar Shahab, Golriz Rostami

**Affiliations:** a Department of Endodontics, Dental School, Shahed University, Tehran, Iran; b Department of Oral and Maxillofacial Radiology, Dental School, Shahed University, Tehran, Iran; c Endodontist, Private Practice

**Keywords:** Apical Zipping, CBCT, Centering Ability, Cone-Beam Computed Tomography, Ledge, Reciproc, Root Canal Preparation, Canal Transportation, Twisted File

## Abstract

**Introduction:** The purpose of this *in vitro* study was to compare the canal transportation and centering ability of Twisted File (TF) to that of Reciproc system. **Methods and Materials:** Forty noncalcified roots with mature apices, minimum length of 19 mm and an apical curvature of 15-30 degrees (according to Schneider’s method), from freshly extracted mandibular and maxillary teeth, were selected for this study. Samples were randomly divided into two groups (*n*=20) and canal preparation with either TF or Reciproc was performed according to manufacturers' instruction. Pre- and post-instrumentation cone-beam computed tomography (CBCT) images were captured and the extent of canal transportation and centering ability of the files were calculated, using the NNT Viewer software and Photoshop CS5, at levels of 3, 4, and 5 mm from the apex. The Mann-Whitney U test was used to analyze the statistical significance between the two groups. **Results:** One fracture occurred in the TF group. TF produced more transportation than Reciproc in both mesiodistal and buccolingual directions; however, the difference between the two systems were not statistically significant except for the TF group at 5-mm distance from the working length, where the difference was significant (*P*>0.05). **Conclusion:** Both file systems were able keep the original curvature of the canal and thus can be considered safe for clinical application.

## Introduction

The main goal of root canal preparation is to clean the root canal system while maintaining the original shape of the canal(s). Achieving this goal can facilitate effective irrigation, root canal medication, and finally, the three dimensional obturation [1]. All instruments and instrumentation techniques have a tendency to transport and alter the original canal shape, especially when the curvature is prominent and being negotiated for the first time [2].

Development of nickel-titanium (NiTi) rotary instruments has provided easier and faster canal instrumentation and has minimized the procedural errors such as ledge, zip, canal transportation and stripping [3]. Many manufacturers have incorporated different designs into their ﬁle systems to minimize apical transportation and achieve faster and more predictable canal preparation [4].

Twisted file (TF) (SybronEndo, Orange, CA, USA) is a rather new rotary system with a triangular cross section. At first this system was available in one size with variable tapers (#25/0.04, 0.06, 0.08, 0.10 and 0.12) and later, sizes 30/0.06, 35/0.06, 40/0.04 and 50/0.04 were added to the system. The files are also available in small assorted (S-ASTD; size 25 with 0.04, 0.06 and 0.08 tapers) and large assorted (L-ASTD; size 25 with 0.06, 0.08 and 0.10 tapers) packages which must be used at speed of 500-625 rpm and the torque of 400 Ncm [5]. TF production implements a specific R-phase heat treating which allows twisting of the NiTi wire. This proprietary technology is used to optimize the molecular phase and properties of NiTi. Therefore, the resulting crystalline structure modification, which has been shown to be better than traditionally processed materials, maximizes the file flexibility and resistance to fracture [6].

Recently, a single-file system known as Reciproc (VDW, Munich, Germany) has been developed with an S-shaped cross-section, a non-cutting tip and sharp cutting edges which implements 150 degrees counterclockwise and then 30 degrees 

clockwise rotation with a speed of 300 rpm [7]. Although it is claimed that by this system there is no need for making a glide-path prior to instrumentation [8], providing a glide-path with an ISO size 10 or 15 hand file is recommended as well [9]. This single file system is available at three different sizes and tapers; 25/0.08, 40/0.06 and 50/0.05 [7]. 

Some studies showed that NiTi instruments were safer with a reciprocating movement for root canal preparation compared to continuous rotary motion [10, 11]. Moreover, this reciprocating motion can lower the chance of file fracture [12]. The reciprocating movement also increases the cyclic fatigue life of the instruments compared to the conventional rotation [10]. There is limited information regarding the influence of reciprocating motion on canal transportation and centering ability compared to continuous rotation.

The present *in vitro* study aimed to compare the canal transportation and centering ability of the two aforementioned instruments, *i.e.* TF and Reciproc, by means of cone-beam computed tomography (CBCT).

## Methods and Materials

This study was conducted on forty mesiobuccal canals from mesial roots of mandibular molars and mesiobuccal roots of maxillary molars with at least 19 mm length, apical curvature of 15-30 degrees (according to Schneider’s method), mature apices and uncalcified canals [12, 13]. After disinfection with 5.25% sodium hypochlorite (NaOCl), samples were stored in 10% formalin solution before experiment. The access cavity was made with a #4 high speed round carbide bur (Dentsply Maillefer, Ballaigues, Switzerland). Then the canal orifices were negotiated. If a #15 K-file could be easily fed into the root canal, the sample was excluded from the study. For establishing the working length (WL), a size 10 K-file (Dentsply, Maillefer, Ballaigues, Switzerland) was placed into the canal until it was visible at the apical foramen. The WL was determined 0.5 mm short of this length and the reference points were marked with an indelible marker on the teeth.

For easier placement of the teeth, the other roots were amputated at the furcation level. At this point, samples were fitted in the desired position by using a silicon-based impression material (Speedex, Coltene/Whaledent, Altstätten, Switzerland). The teeth were inserted in parallel positions to the wall of the plastic mold so that the most apical point of the roots touched the base of the mold. CBCT images were captured, both before and after instrumentation, in the same position.

The teeth were randomly divided into the following two groups (*n*=20): In Group A, regarding no need for creation of a glide-path, the root canals were prepared with the Reciproc file size 25/0.08 (VDW, Munich, Germany) installed on a 1:16 gear rotary handpiece, powered by an electric torque-controlled motor (Silver; VDW, Munich, Germany), at a pre-programmed movement format as “Reciproc” mode. Feeding the Reciproc files into the canals was done with a slow in-and-out pecking motion. After every three in-and-out movements, the file was pulled out for cleaning its flutes and canal irrigation with normal saline. The instrument was then reinserted in the same manner until the established working length was reached. After completion of the canal preparation, teeth were scanned with CBCT in the same manner used for the initial scans.

**Figure 1:**
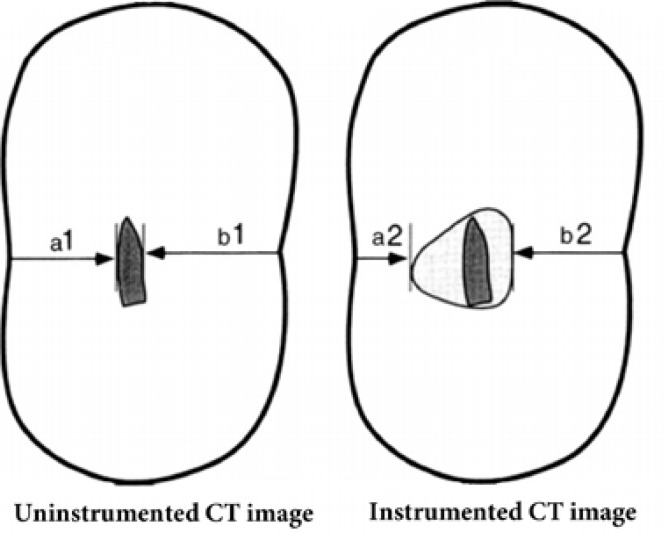
Schematic shape of tooth sections showing the transportation and centering ratios; degree of changes were calculated by (a1-a2)-(b1-b2)

In Group B, the samples were prepared using TF system (SybronEndo, Orange, CA, USA) coupled to a 1:16 reduction gear rotary handpiece driven by the same electric device with a speed of 500 rpm and the torque of 400 Ncm, in a crown-down manner. The first file, 25/0.06, was primarily used in a passive manner and 2 mm short of the WL for shaping the coronal one- or two-thirds of the canal. Then the other files *i.e.* 25/0.04, 25/0.06 and 25/0.08 were respectively used to the WL [14]. In this way the final apical size and canal taper was 25/0.08.

The same operator prepared the samples of both groups and each rotary file was discarded after preparation of four canals. Canals were lubricated using an EDTA-containing gel (MD Chelcream, Meta Biomed Co. Ltd, Chungbuk, Korea). Recapitulation and irrigation with 2 mL of 5.25% NaOCl was done after the use of each instrument. Teeth were scanned before and after instrumentation with their roots being perpendicular to the beam of the CBCT device (NewTom VGi, QR SRL Co., Verona, Italy) with the following settings: 110 KV, 9.5 mA, a 0.125-mm voxel size, and a 0.125-mm axial thickness [15, 16]. Calculation and comparison of all scans were made at 3, 4 and 5 mm from the apical foramen using the software NTT Viewer (NTT Software Corporation, Yokohama, Japan) and Adobe Photoshop CS5 (Adobe Systems Inc., San Jose, CA) [16].

**Table1:** Transportation in the defined levels (MD=mesiodistal, BL=buccolingual, WL=working length)

**Rotary**	**Distance from WL**	**MD transportation Mean (SD)**	**BL transportation Mean (SD)**
**Reciproc**	3 mm	0.08 (0.09)	0.09 (0.75)
**TF**	3 mm	0.13 (0.14)	0.11 (0.11)
**Reciproc**	4 mm	0.09 (0.09)	0.09 (0.68)
**TF**	4 mm	0.12 (0.17)	0.09 (0.07)
**Reciproc**	5 mm	0.04 (0.06)	0.13 (0.08)
**TF**	5 mm	0.14 (0.10)	0.14 (0.10)

In the next step scan images of same sections were superimposed on each other and the degree of changes in mesiodistal and buccolingual dimensions was recorded separately with Adobe Photoshop software CS5 according to the following formula: (a1-a2)-(b1-b2), where a1 is the shortest distance from the mesial (or lingual) aspect of non-instrumented canal to the mesial edge of the root, and a2 is the shortest distance between the mesial (or lingual) edge of instrumented canal to the mesial (or lingual) edge of the root (b1 and b2 are defined similarly as shown in Figure 1) [17]. According to this formula, the result 0 indicates that no canal transportation has occurred, negative result is the indicator of distal (or buccal) transportation, and positive result is representative of mesial (or lingual) transportation.

Centering ability was calculated using the ratio of a1-a2/b1-b2, or b1-b2/a1-a2 (the lower value is set as standard for the statistical evaluation). In this formula, the value of 1 indicates complete centering, and the results other than 1 show a change in the original canal axis [17] .

The distribution of the obtained data was analyzed by One-Sample Kolmogorov-Smirnov Test. This test showed that the data points did not pass the normality test and the distribution of the data obtained by this study did not follow a Gaussian pattern. Therefore, we used the distribution free (non parametric) Mann-Whitney U test to compare the two groups in both buccolingual and mesiodistal dimensions. The SPSS software (SPSS version 17.0, SPSS, Chicago, IL, USA) was used for the statistical analysis and a *P*-value equal or less than 0.05 was considered a significant difference.

## Results

**Figure 2:**
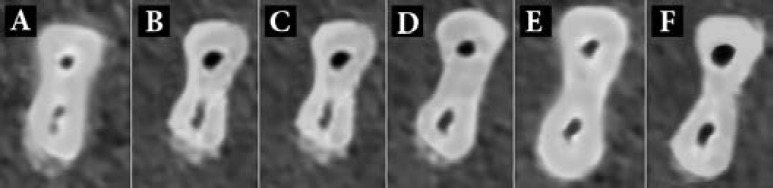
The scan images at *A *and* B)* 3 mm; *C *and* D)* 4 mm and; *E *and* F)* 5 mm from the apical before and after preparation with Reciproc

**Figure 3:**
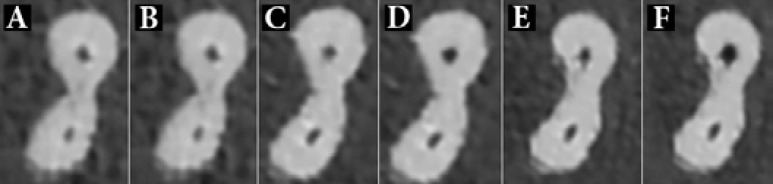
The scan images at *A* and *B)* 3 mm; *C* and *D)* 4 mm; and E and *F)* 5 mm from the apical before and after preparation with Twisted File

The mean±SD for mesiodistal and buccolingual transportation values in both systems, are shown in Table 1. Also Figures 2 and 3 show the CBCT images at 3, 4 and 5-mm distances from the apical for Reciproc and TF file systems.

During this study, there was one fracture in the TF group during the use of a 25/0.08 file. According to the mean degree of transportation and centering ability in each section, TF produced more transportation compared to Reciproc in both mesiodistal and buccolingual directions. However, the difference between the two systems was only statistically significant in the 5-mm distance from the apex. In mesiodistal dimension, the Reciproc system showed significantly lower transportation (*P*=0.010) and better centering ability (*P*=0.028). On the other hand, the difference between the two groups at 3 or 4-mm distance from apex did not reach the level of significance.

## Discussion

There are many devices with automated systems that can be implemented by the dentist or the endodontist for root canal preparation. The use of these systems help overcome or reduce many clinical problems and also saves time, reduces operator’s fatigue and occurrence of canal alterations such as zips, apical or lateral perforations and elbow formation [18].

Transportation is defined as the undesired deviation of canal's original shape to a new iatrogenic location of the external exit of the canal. Different types of transportation may happen during the root canal therapy, and only the type I transportation could be treated non-surgically [19]. Difficulty in getting back to the original shape, leads to insufficient cleaning and shaping and over-reduction of radicular dentin in one or two of the canal walls [20]. Eventually, apical transportation may lead to zipping or perforation of the canal [21]. Apical transportations that are more than 0.3 mm can jeopardize the outcome of treatment due to the significant decrease in the sealing ability of root filling material [22].

Among different methods described in the literature for the evaluation of shaping ability of various instruments and preparation techniques, CBCT imaging is one of the latest innovations that provide detailed three-dimensional observations at a low radiation dose. In addition because of the possibility of choosing smaller field of view (FOV) compared to the medical CT scans, the resulting images have higher resolutions, and therefore are more accurate and have a higher diagnostic capability [23, 24]. In the present study the CBCT device was set at resolution of 0.125 µm, which is better than similar previous studies, and also has more accuracy in determining the smallest changes of canal anatomy compared to the previous studies [25-27]. CBCT offers many advantages, although micro-CT remains the gold standard for evaluating the centering ability of different file systems. 

A number of different *in vitro* models are described in the literature, among which the resin block was advocated for reproducibility and calibration of the experimental design [28]. Regarding the difference between the hardness of dentine and resin, it is shown that the dentine microhardness in areas close to the pulp space was two times more than that of resin blocks.

Therefore, a double force must be applied to remove the dentine. Moreover, the size of resin chips and their dryness can result in the blockages of the canal and this can produce problems for deeper penetration of the instrument. Thus, the result of this kind of study could not be applicable in the clinical practice at this stage [29]. Root canals of extracted human mandibular molars were implemented in the present study because apart from stimulating the clinical conditions, they usually present a prominent curvature as well as a mesiodistal flattening.

The results of this study show that, except for the significant superiority of Reciproc at 5-mm distance from the apex, both systems produce similar outcomes in terms of centering ability and canal transportation. In other words, both systems provided relatively centered preparations and maintained the original shape of the curved canal with minimal changes. These results are supported by previous studies that have used the TF system [30, 31]. This similarity can be explained due to the fact that both systems have been produced by modifications in the transitional R-phase of NiTi alloy. In other words, TF is produced by twisting the NiTi wire in this phase while Reciproc has been undergone compositional and thermo-mechanical changes leading to a novel NiTi alloy named M-wire [8]. The special process of production of these file systems offers superior flexibility and increased resistance to cyclic fatigue when compared to the traditional NiTi rotary systems that are produced by grinding process. TF engages into root canal by the shaving action of a positive 45^°^ rake angle, whereas the progression of Reciproc in the canal depends on the counterclockwise and clockwise movements [8].

According to the investigation by Burklein *et al.*, canal preparation with Reciproc system was significantly faster. It also achieved better results in apical third in terms of cleansing compared to the other single-file system (WaveOne, Dentsply Maillefer, Ballaigues, Switzerland) and was also faster than rotary systems with multiple files [8]. Although we did not analyze the issue of time as a variable in this study; it seems that canal preparation with Reciproc single-file system, is faster and easier than a multiple-file system. Investigations showed that Reciproc files have a continuous taper over the first 3 mm of their working part followed by a decreasing taper until the shaft [8].

The concept of preparing the root canal without creating a glide-path by means of K-files sizes 10 or 15, seems like a new concept in this area. Creating a glide-path is mandatory before using NiTi rotary file system for preventing their sticking into the canal. Our study showed that creating such a glide-path is not an essential factor [8].

No file failure occurred in Reciproc group and this can be attributed to the special reciprocating movement and S-shaped cross section of this system. Reciprocation reduces the possibility of fracture caused by taper lock or torsional fracture; therefore, using this system seems to be safer than TF or other similar rotary systems. In the Reciproc system, one instrument is preparing the entire root canal system instead of using several files with different sizes and tapers. Therefore, the file would be prone to more fatigue than usual, which is the reason for discarding the instrument after each time of application. In addition, Reciproc files are designed as a single used system, so the cross-contamination of reiterated usage would be eliminated. Moreover, the inability of usual techniques to adequately clean and sterilize instruments from prions, dentin particles and organic debris could be prominent [32, 33]. Besides, dentinal deposits are reported to play an essential role in the clinical failure of rotary instruments [34].

Some investigators believe that the constant revolution of the file toward the outer part of the curve (especially in prominent curved root canals) results in minimal shaping of the inner part of the curve. The reciprocating movement prepared the canal centered on the original shape, which means that it has enlarged almost equally in both inner and outer directions [4].

The difference observed in the transportation at 5-mm distance from the apex can be attributed to the higher number of instruments used from crown to the apex in TF group in contrast to the Reciproc single-file system.

## Conclusion

This *in vitro* study revealed that Twisted File and Reciproc systems do not differ significantly in terms of canal centering ability and transportation.

## References

[1] Ruddle CJ, Cohen S, Bums RC (2002). Cleaning and shaping the root canal system.

[2] Griffiths IT, Bryant ST, Dummer PM (2000). Canal shapes produced sequentially during instrumentation with Quantec LX rotary nickel-titanium instruments: a study in simulated canals. Int Endod J.

[3] Parashos P, Messer HH (2006). Rotary NiTi instrument fracture and its consequences. J Endod.

[4] Franco V, Fabiani C, Taschieri S, Malentacca A, Bortolin M, Del Fabbro M (2011). Investigation on the shaping ability of nickel-titanium files when used with a reciprocating motion. J Endod.

[5] Aminsobhani M, Ghorbanzadeh A, Bolhari B, Shokouhinejad N, Ghabraei S, Assadian H, Aligholi M (2010). Coronal microleakage in root canals obturated with lateral compaction, warm vertical compaction and guttaflow system. Iran Endod J.

[6] Plotino G, Grande NM, Testarelli L, Gambarini G (2012). Cyclic fatigue of Reciproc and WaveOne reciprocating instruments. Int Endod J.

[7] http://www.vdw-dental.com/en/service/%20information/catalogues-and-brochures.html.

[8] Burklein S, Hinschitza K, Dammaschke T, Schafer E (2012). Shaping ability and cleaning effectiveness of two single-file systems in severely curved root canals of extracted teeth: Reciproc and WaveOne versus Mtwo and ProTaper. Int Endod J.

[9] Shahravan A, Rekabi A, Shahabi H, Ashuri R, Mirzazadeh A, Rad M, Haghani J (2010). A digital stereomicroscopic study of the furcation wall thickness of mesiobuccal roots of maxillary first and second molars. Iran Endod J.

[10] De-Deus G, Moreira EJL, Lopes HP, Elias CN (2010). Extended cyclic fatigue life of F2 ProTaper instruments used in reciprocating movement. Int Endod J.

[11] Varela-Patiño P, Martín-Biedma B, Rodriguez-Nogueira J, Cantatore G, Malentaca A, Ruiz-Pinón M (2008). Fracture rate of nickel-titanium instruments using continuous versus alternating rotation. Endodontic Practice Today.

[12] Tsesis I, Amdor B, Tamse A, Kfir A (2008). The effect of maintaining apical patency on canal transportation. Int Endod J.

[13] Schneider SW (1971). A comparison of canal preparations in straight and curved root canals. Oral Surg Oral Med Oral Pathol.

[14] Gambarini G, Plotino G, Grande NM, Al-Sudani D, De Luca M, Testarelli L (2011). Mechanical properties of nickel-titanium rotary instruments produced with a new manufacturing technique. Int Endod J.

[15] Özer SY (2011). Comparison of root canal transportation induced by three rotary systems with noncutting tips using computed tomography. Oral Surg Oral Med Oral Pathol Oral Radiol Endod.

[16] Oliveira CA, Meurer MI, Pascoalato C, Silva SR (2009). Cone-beam computed tomography analysis of the apical third of curved roots after mechanical preparation with different automated systems. Braz Dent J.

[17] Short JA, Morgan LA, Baumgartner JC (1997). A comparison of canal centering ability of four instrumentation techniques. J Endod.

[18] Guelzow A, Stamm O, Martus P, Kielbassa AM (2005). Comparative study of six rotary nickel-titanium systems and hand instrumentation for root canal preparation. Int Endod J.

[19] Gluskin AH, Peters CI, Wong RDM, Ruddle GJ, Ingle JI, BaKland LK, Baumgartner JG (2006). Retreatment of Non-Healing Endodntic Therapy and Management of Mishaps. Ingles Endodontics.

[20] Paqué F, Musch U, Hülsmann M (2005). Comparison of root canal preparation using RaCe and ProTaper rotary Ni-Ti instruments. Int Endod J.

[21] Karabucak B, Gatan AJ, Hsiao CK, Iqbal MK (2010). A comparison of apical transportation and length control between EndoSequence and Guidance rotary instruments. J Endod.

[22] Wu MK, Fan B, Wesselink PR (2000). Leakage along apical root fillings in curved root canals Part I: effects of apical transportation on seal of root fillings. J Endod.

[23] Ludlow JB, Davies-Ludlow LE, Brooks SL, Howerton WB (2006). Dosimetry of 3 CBCT devices for oral and maxillofacial radiology: CB Mercuray, NewTom 3G and i-CAT. Dentomaxillofac Radiol.

[24] Hatcher DC (2010). Operational principles for cone-beam computed tomography. J Am Dent Assoc.

[25] Loizides AL, Kakavetsos VD, Tzanetakis GN, Kontakiotis EG, Eliades G (2007). A comparative study of the effects of two nickel-titanium preparation techniques on root canal geometry assessed by microcomputed tomography. J Endod.

[26] Yang G, Yuan G, Yun X, Zhou X, Liu B, Wu H (2011). Effects of two nickel-titanium instrument systems, Mtwo versus ProTaper universal, on root canal geometry assessed by micro-computed tomography. J Endod.

[27] Mahran AH, AboEl-Fotouh MM (2008). Comparison of effects of ProTaper, HeroShaper, and Gates Glidden burs on cervical dentin thickness and root canal volume by using multislice computed tomography. J Endod.

[28] Lim KC, Webber J (1985). The validity of simulated root canals for the investigation of the prepared root canal shape. Int Endod J.

[29] Hülsmann M, Peters OA, Dummer PMH (2005). Mechanical preparation of root canals: shaping goals, techniques and means. Endodontic Topics.

[30] Gergi R, Rjeily JA, Sader J, Naaman A (2010). Comparison of canal transportation and centering ability of twisted files, Pathfile-ProTaper system, and stainless steel hand K-files by using computed tomography. J Endod.

[31] El Batouty KM, Elmallah WE (2011). Comparison of canal transportation and changes in canal curvature of two nickel-titanium rotary instruments. J Endod.

[32] Schneider K, Korkmaz Y, Addicks K, Lang H, Raab WH (2007). Prion protein (PrP) in human teeth: an unprecedented pointer to PrP's function. J Endod.

[33] Sonntag D, Peters OA (2007). Effect of prion decontamination protocols on nickel-titanium rotary surfaces. J Endod.

[34] Alapati SB, Brantley WA, Svec TA, Powers JM, Nusstein JM, Daehn GS (2004). Proposed role of embedded dentin chips for the clinical failure of nickel-titanium rotary instruments. J Endod.

